# Limitations of the Senses

**Published:** 1878-06

**Authors:** George M. Beard


Limitations of the Senses.—The senses, which have hitherto been regarded as infallible, are even more narrowly defined than the memory or the higher qualities of intellect. So narrow is the range of vision—and the sight is certainly the best of the five senses—that the retina can appreciate a few only of the rays that come from the sun. The vibrations of ether beyond the red at one end of the spectrum, and the violet at the other are of no value in vision, ethereal undulations higher than seven hundred and ninety trillions a second, or lower than four hundred trillions a second, being powerless to affect it. The senses, indeed, are not formed to enable man to solve the problem of Nature, but as with the lower animals, merely to make existence possible, and in a limited and incidental way, agreeable. And yet it is through these feeble senses that all human knowlege enters the brain, since all deductive reasoning must be based on previous inductive observations.—Dr. George M. Beard, *in Popular Science Monthly for June.*

